# Amelioration of Acute Oxazolone-Induced Colitis via Oral Administration of EPICERTIN, a Mucosal Healing Biotherapeutic for Inflammatory Bowel Disease

**DOI:** 10.3390/biomedicines14081777

**Published:** 2026-08-06

**Authors:** Wendy M. Kittle, Micaela A. Henderson, Noel Verjan Garcia, Hong Li, Katarina L. Mayer, Jimmy F. Cifuentes Jimenez, Kelly M. Lee, Kavitha Yaddanapudi, Nobuyuki Matoba

**Affiliations:** 1 Department of Pharmacology and Toxicology, School of Medicine, University of Louisville, Louisville, KY 40292, USA; wendy_kittle@outlook.com (W.M.K.); hendersonm@hanover.edu (M.A.H.); katarina.mayer@louisville.edu (K.L.M.); jimmy.cifuentes@louisville.edu (J.F.C.J.); 2 Center for Predictive Medicine, School of Medicine, University of Louisville, Louisville, KY 40202, USA; nverjan@gmail.com; 3 Brown Cancer Center, School of Medicine, University of Louisville, Louisville, KY 40202, USA; hong.li@louisville.edu (H.L.); kavitha.yaddanapudi@louisville.edu (K.Y.); 4 Department of Chemical Engineering, JB Speed School of Engineering, University of Louisville, Louisville, KY 40292, USA; kelly.lee.1@louisville.edu; 5 Department of Surgery, School of Medicine, University of Louisville, Louisville, KY 40202, USA

**Keywords:** Inflammatory bowel disease, ulcerative colitis, epithelial restitution, mucosal healing, EPICERTIN, biotherapeutic, oxazolone colitis, preclinical efficacy, imaging mass cytometry, CyTOF

## Abstract

**Background/Objectives**: Ulcerative colitis (UC) is a chronic, relapsing inflammatory bowel disease (IBD) lacking therapies that directly promote mucosal healing, an important clinical endpoint and therapeutic goal for achieving remission and improved long-term outcomes. Epithelial restitution is a key component of effective mucosal healing. EPICERTIN, a novel biotherapeutic candidate, has previously demonstrated epithelial repair activity in dextran sulfate sodium (DSS)-induced colitis models and enhanced epithelial cell viability in human IBD colon explants. To further substantiate its therapeutic efficacy in a UC-relevant context, we evaluated EPICERTIN in acute oxazolone (OXA)-induced colitis in BALB/c mice, a model reflecting the adaptive immune-driven inflammation and histopathologic characteristics of human UC. **Methods**: Orally administered EPICERTIN, at escalating doses (0.3 µg, 3 µg, and 30 µg), was assessed for therapeutic efficacy in male and female mice through body weight, Disease Activity Index (DAI) scores, and histopathology. Wound healing and immune impacts were examined using qRT-PCR, Imaging Mass Cytometry (IMC), and Cytometry by Time of Flight (CyTOF) in male mice. **Results**: EPICERTIN effectively mitigated acute OXA-induced colitis. The 3 µg dose provided the greatest benefit, improving body weight recovery and reducing DAI and histopathological damage scores. Treatment reduced *Il1b* while increasing *Cdh1* expression, accompanied by higher levels of epithelial markers (pan-cytokeratin (PanCK), E-cadherin, epithelial cell adhesion marker (EpCAM)) and decreased fibrotic markers (collagen type I, alpha-smooth muscle actin (α-SMA), fibronectin). Moreover, EPICERTIN reduced inflammatory lymphoid and myeloid cells while increasing γδ T cells in the colon lamina propria. **Conclusions**: Collectively, these results demonstrate that EPICERTIN promotes epithelial restitution, suppresses inflammation, and supports mucosal healing, substantiating its therapeutic potential for UC.

## 1. Introduction

Inflammatory bowel disease (IBD), encompassing Crohn’s disease (CD) and ulcerative colitis (UC), is a chronic and relapsing condition marked by persistent inflammation of the gastrointestinal (GI) tract. CD manifests as non-sequential transmural inflammation anywhere in the GI tract, while UC predominantly affects the inner layers of the distal GI tract in a continuous manner, typically beginning at the rectum and extending into the distal colon [1,2]. The etiology of IBD is complex and multifactorial, involving a dysregulated immune response, genetic susceptibility, and environmental factors [1,3]. In UC, patients often experience a range of symptoms, including severe abdominal pain, chronic diarrhea, and fatigue, significantly impacting their quality of life and potentially necessitating surgical intervention to manage complications or achieve disease control when medical therapies fail [2,4]. Current IBD therapeutic approaches primarily focus on controlling inflammation, but treatments such as aminosalicylates, corticosteroids, immunomodulators, and biologics targeting inflammatory cytokines or integrins can lose efficacy over time and often have adverse side effects [4,5,6]. In many cases, these therapies fail to achieve the most important clinical UC endpoint: mucosal healing. This process of wound repair and tissue regeneration is critical for achieving lasting remission and improving long-term patient outcomes [3,7,8], yet no Food and Drug Administration (FDA)-approved agents specifically target it. Because epithelial barrier defects and impaired epithelial regeneration are hallmarks of IBD, particularly UC, agents that promote epithelial wound repair may address a major unmet clinical need in UC treatment [9,10].

Our research is focused on the preclinical development of a novel biotherapeutic agent, EPICERTIN (Epithelial Cell ER-Targeted Protein), which is designed to fill this unmet need. EPICERTIN is a recombinant variant of the nontoxic B subunit of cholera toxin (CTB) [11] and represents a promising candidate for the treatment of inflammatory mucosal disorders like IBD. Its mechanism of action begins with binding to GM1 ganglioside on the cell surface, which facilitates retrograde transport to the endoplasmic reticulum (ER) [12]. Within the ER, EPICERTIN’s unique C-terminal ER-retention motif (KDEL) prolongs its residence time via interaction with KDEL receptors (KDELRs), inducing an adaptive unfolded protein response (UPR) that activates wound healing pathways [12]. In addition, KDELR-mediated signaling may enhance vesicular trafficking [13]. Collectively, these responses promote cell survival and re-epithelialization, which leads to mucosal healing.

EPICERTIN’s therapeutic potential has been demonstrated in ex vivo human IBD explants and in both acute and chronic dextran sulfate sodium (DSS)-induced colitis models [12,14,15]. While these models enabled investigation of EPICERTIN’s effects on epithelial injury and repair, they do not fully capture the multifaceted nature of human IBD, particularly the complex adaptive immune responses that play a central role in disease pathogenesis [16]. For this reason, therapeutic candidates for IBD are typically evaluated in multiple colitis models to comprehensively assess their efficacy. To further define EPICERTIN’s therapeutic breadth, we investigated its effects in the acute oxazolone (OXA) colitis model. OXA is a haptenating agent that induces a skewed T helper type 2 (Th2)-driven adaptive immune response against colonic epithelial and luminal antigens in BALB/c mice [16,17,18,19]. This model is widely employed for its ability to recapitulate key immunological and histopathological features of human UC, such as damage to the mucosa and submucosa with mixed inflammatory cell infiltration (neutrophils, macrophages, and lymphocytes) [16,19]. Given EPICERTIN’s efficacy in human IBD explants and DSS colitis, we hypothesized it would exhibit significant therapeutic effects against hapten-induced tissue damage and inflammation.

Our results indicate that oral EPICERTIN treatment effectively mitigates acute OXA-induced colitis, with the 3 µg dose demonstrating the greatest therapeutic benefit. This finding is particularly important as it provides novel evidence for EPICERTIN’s efficacy in a model reflecting adaptive immune-driven intestinal inflammation, a characteristic of many IBD presentations. These results suggest that EPICERTIN may confer broad protective effects against diverse forms of epithelial injury and intestinal inflammation, reinforcing its potential as a therapeutic candidate for IBD.

## 2. Materials and Methods

### 2.1. EPICERTIN Production

EPICERTIN was expressed in BL21 (DE3) *Escherichia coli* and purified using immobilized metal affinity chromatography (IMAC) followed by ceramic hydroxyapatite (CHT) Type I, based on a previously established protocol for CTB [20]. Minor modifications were made to optimize the process, including the use of Terrific Broth (TB) medium, which increased the yield by 50%. The composition of the purification buffers was also adjusted for optimal purification, as follows: IMAC buffer A (10 mM Tris HCl, 300 mM sodium chloride, pH 8.0); IMAC buffer B (10 mM Tris HCl, 300 mM sodium chloride, 250 mM imidazole); CHT buffer A (10 mM Tris HCl, 5 mM sodium phosphate, 250 mM sodium chloride, pH 8.0); and CHT buffer B (10 mM Tris HCl, 200 mM sodium phosphate, 250 mM sodium chloride, pH 8.0). Following buffer exchange to phosphate-buffered saline (PBS) pH 7.4, via a 3K Amicon Ultra centrifugal filter (Millipore, Burlington, MA, USA), the final product was endotoxin removed to a level of <0.1 Eu/mg and assessed for purity (SDS-PAGE, size exclusion chromatography high-performance liquid chromatography (SEC-HPLC)), identity (anti-CTB Western blot), and potency (GM1-binding/KDEL-detection ELISA), as previously described [21].

### 2.2. Animal Studies, Ethics, Housing, and Husbandry

General animal care and housing procedures were conducted in accordance with the Guide for the Care and Use of Laboratory Animals, the Animal Welfare Act and associated regulations, and institutional standards. All animal studies were approved and conducted under the oversight of the University Committee for Animal Welfare (UCAW), formerly Institutional Animal Care and Use Committee (IACUC), protocol #23247. Mice were acclimatized for seven days before study initiation and maintained on a 12 h light/dark cycle with ad libitum access to standard rodent chow and water. Environmental enrichment was provided in accordance with institutional husbandry practices.

### 2.3. Oxazolone Colitis Induction and Treatment

Acute colitis was induced using oxazolone (OXA; Sigma-Aldrich, St. Louis, MO, USA) according to an established protocol [17], with slight modification. Briefly, eight-week-old male (*n* = 8 per group) and female BALB/c mice (*n* = 8 per group) were obtained from Charles River Laboratories (Wilmington, MA, USA); mice were wild-type and experimentally naïve before OXA sensitization and challenge. Following a one-week acclimatization period, mice were allocated to treatment groups to achieve comparable mean baseline body weights; formal randomization was not used. Treatments were administered within a consistent time window using the same group sequence on dosing days, and cages remained in consistent rack locations throughout the study. Group allocation was known during animal assignment and treatment administration.

Mice were presensitized via topical application of 3% OXA in 100% ethanol to shaved abdominal skin. Seven days later, colitis was induced by intrarectal challenge with 1% OXA in 50% ethanol. Mice received oral doses of PBS, 0.3 µg, 3 µg, or 30 µg EPICERTIN on Day 0 (challenge day) and Day 3, with all mice receiving prerequisite oral administration of sodium bicarbonate (Sigma-Aldrich, St. Louis, MO, USA) to neutralize stomach acid.

Body weight and clinical condition were assessed daily and before dosing. Animals were observed for clinical signs of morbidity or distress, including changes in appearance, activity, posture, respiration, grooming, and stool characteristics. To support food intake and hydration during acute colitis, wet chow was provided to all animals throughout the study. Supportive perianal care, including gentle cleansing with warm water when needed, was provided to maintain comfort and facilitate normal stool passage. Humane endpoints were predefined as a DAI score of 4 or ≥20% body-weight loss, whichever occurred first; animals meeting either criterion were humanely euthanized according to UCAW protocol guidelines. Animals with <5% body-weight loss one day after challenge were excluded a priori because this indicated inadequate disease induction. All remaining animals were euthanized two days after the second dose by carbon dioxide asphyxiation followed by cervical dislocation, in accordance with UCAW protocol guidelines. Final analyzed group sizes ranged from *n* = 6–8 per group, as specified in the figure legends. Across all OXA-induced colitis studies reported herein, including the complementary MLN study presented in Figure S1, a total of 99 mice were enrolled. Group sizes were informed by anticipated attrition associated with severe disease induction and predefined exclusion criteria. For all in vivo experiments, the individual mouse was considered the experimental unit.

OXA colitis was induced in both male and female mice to confirm initial therapeutic efficacy. Treatment effects were directionally consistent between sexes, with 3 μg EPICERTIN producing the greatest benefit in both; sex-stratified summaries of body weight, DAI and histopathological score are provided in Supplementary Figures S2 and S3. In males, treatment effects reached statistical significance for body weight recovery, DAI and histological damage score at the time of sacrifice. In females, body weight loss was significantly reduced with 0.3 μg and 3 μg during the acute phase, but treatment effects were no longer significant at euthanasia because PBS-treated female mice recovered spontaneously, leaving limited dynamic range for detecting a treatment benefit at that timepoint. The study was not powered to evaluate sex as a biological variable, and the absence of significant differences at individual timepoints should not be interpreted as evidence of equivalence. Because measurable disease persisted at the sacrifice timepoint in males but had largely resolved in PBS-treated females, male cohorts provided the window in which treatment-associated differences in immune composition could be assessed; subsequent imaging mass cytometry (IMC) and cytometry by time of flight (CyTOF) studies were therefore conducted in male cohorts.

### 2.4. Histological Analysis

Colons were collected and thoroughly washed in ice-cold PBS. The colons were then prepared as Swiss-rolls by first making a longitudinal incision. Tissues were then rolled from the distal to the proximal end, fixed in 10% neutral buffered formalin (VWR, Radnor, PA, USA), dehydrated in 70% ethanol, and subsequently paraffin embedded, sectioned (10 μm), and stained with hematoxylin and eosin (H&E). Histopathological damage was scored blinded to treatment-group allocation on the H&E-stained sections and adapted from previously described methods [22,23] (Table S1).

### 2.5. Gene Expression (qRT-PCR)

To characterize the transcriptional profile of the colonic response, three animals per group were allocated from the male cohort for dedicated gene expression analysis, separate from those allocated for histological assessment. Selection of animals for gene-expression analysis was performed with knowledge of treatment-group allocation. These animals had DAI scores closest to the respective group mean to provide a representative assessment of therapeutic impact. Sample processing and analysis were conducted using the same predefined workflow across all samples.

Distal colon tissue (~14 mg) was homogenized in QIAzol, and total RNA purified using the RNeasy Microarray Tissue Kit (Qiagen, Hilden, Germany). RNA quality and concentration were verified spectrophotometrically (Thermo Scientific™ NanoDrop™ OneC, Thermo Fisher Scientific, Waltham, MA, USA). First-strand cDNA was synthesized from 500 ng RNA (RT^2^ Easy First Strand cDNA Synthesis Kit, Qiagen, Hilden, Germany). qRT-PCR was performed on a Quant Studio 3™ PCR System (Applied Biosystems, Thermo Fisher Scientific, Waltham, MA, USA) using Qiagen RT^2^ SYBR Green ROX Master Mix and RT^2^ Profiler PCR Array Plates. Probes included target genes (*Cdh1*, *Tgfb1*, *Wnt5a*, *Col1a2*, *Col3a1*, *Col4a1*, *Mmp2*, *Ctsk*, *Ctsg*, *Csf3*, *Tagln*, *Angpt1*, *Il6*, *Il10*, *Il1b*, *Cxcl3*, *Ccl7*, *Serpine1*, *Ifng*) and housekeeping genes (*Actb*, *Gapdh*). The assay was supplied as pre-validated primer sets, validated by the manufacturer for specificity and amplification efficiency, and their sequences are proprietary. Assay identifiers, RefSeq accession numbers, Entrez Gene IDs and amplicon lengths for all targets and reference genes are provided in Supplementary Table S2; primer sequences are available from the manufacturer on request. Cycle threshold (Ct) values and expression ratios were derived using Thermo Fisher Connect Design & Analysis Software (v2.6.2). No animals, tissue samples, or qRT-PCR data points were excluded after selection of the gene-expression cohort.

### 2.6. Imaging Mass Cytometry (IMC)

For IMC analysis, three representative animals were selected from the PBS and 3 µg EPICERTIN groups in the male histology cohort, based on their average DAI and histopathological damage scores. Considering the resource-intensive nature of the IMC platform, this cohort size was chosen for the initial characterization; this number provided a proof-of-concept assessment of cellular orchestration at a high resolution, given the maintained spatial stability of the fixed tissue segments. A pathologist’s consultation was used to select three technical replicate regions of interest (ROIs) per animal, focusing on areas of greatest histological damage for targeted analysis of wounded areas. ROI values were averaged within each animal prior to analysis, and the individual animal was the experimental unit (*n* = 3 per group). Animal and ROI selection for IMC was performed with knowledge of treatment-group allocation. Tissue staining followed Fluidigm’s IMC protocol (PN 400322 A3), including heat-induced epitope retrieval (IHC World, Ellicott City, MD, USA) and overnight incubation with a metal-conjugated antibody cocktail (Table S3). DNA was visualized with Cell-ID™ Intercalator-Ir (Standard BioTools, South San Francisco, CA, USA). Tissues were imaged on a Hyperion Imaging System (Standard BioTools), and image analysis was performed using MCD Viewer (version 1.0.5, Standard BioTools) for data visualization, ilastik (University of Heidelberg, Heidelberg, Germany) for cell segmentation, CellProfiler (Broad Institute, Cambridge, MA, USA) for single cell analysis to produce cell masks, histoCAT (University of Zurich, Zurich, Switzerland) for cell mask conversion to fcs files, followed by FlowJo software (version 10.10, BD Biosciences, Ashland, OR, USA) to analyze cell phenotypes. Cell phenotypes were defined using established markers, and positive cell counts within tissue regions were quantified. IMC gating and quantitative analysis were conducted with knowledge of group allocation because initial gating thresholds were calibrated using control samples. Once established, the same gating strategy and analysis parameters were applied consistently across all samples. No selected animals, ROIs, or IMC-derived data points were excluded from analysis.

For t-SNE generation, all samples were concatenated into a single file, and the gating strategy was applied to this concatenated dataset. A single t-SNE embedding was then computed from the pooled events, so that coordinates are directly comparable across all panels and treatment groups. Each panel displays one parent population with selected subsets overlaid. T-SNE population colors were assigned automatically by the software in gating-hierarchy order along a fixed spectrum. The assignment is a software default rather than a designed encoding: it conveys no quantitative property, and no analysis or conclusion presented here depends on the color of any individual population. These panels are included to illustrate the spatial distribution of annotated populations within each group.

### 2.7. Cytometry by Time of Flight (CyTOF)

Following the same study design, acute OXA colitis was induced in a separate male cohort, and animals were treated with either PBS (*n* = 5) or 3 µg EPICERTIN (*n* = 5). A cohort of *n* = 5 was utilized for CyTOF analysis to ensure sufficient statistical power and accommodate potential cell viability attrition inherent to the isolation of lamina propria lymphocytes. Single-cell suspensions were prepared from the isolation of the colon lamina propria (CLP) following the manufacturer’s instructions of Lamina Propria Dissociation Kit (Miltenyi Biotec, Bergisch Gladbach, Germany). Live cells were counted via trypan blue exclusion (Bio-Rad, Hercules, CA, USA), and samples were then processed with Standard BioTools reagents as described previously [24]. Briefly, cells were stained for viability with cisplatin and subsequently stained with a metal-conjugated antibody cocktail to identify surface and intracellular antigens (Table S4). After staining and fixation, cells were incubated with a Cell-ID™ Intercalator-Ir (Standard BioTools) to label DNA. The samples were then acquired on a Helios CyTOF system (Standard BioTools). FCS files were normalized and analyzed using FlowJo software (version 10.10, BD Biosciences). The unsupervised clustering algorithm, Flow Self-Organizing Map (FlowSOM), implemented as a plugin within FlowJo (BD Biosciences, Ashland, OR, USA), was employed to define and quantify cell phenotypes. These phenotypes were further visualized using t-distributed stochastic neighbor embedding (t-SNE). Clustered populations were assigned putative immune identities by supervised annotation according to their dominant canonical marker-expression patterns (Table S5), with annotation informed by expert immunologic review; clusters without a definitive phenotype were designated unclassified. CyTOF sample processing, acquisition, and downstream analysis were conducted with knowledge of treatment-group allocation; the same preprocessing, FlowSOM clustering, and annotation workflow was applied across all samples. The t-SNE embedding and population color assignments were generated as described in Section 2.6. When corresponding quantitative analyses are shown, the same population colors are retained and explicitly identified to facilitate comparison across panels. No animals, CyTOF samples, or CyTOF-derived data points were excluded from the final analysis.

### 2.8. Statistical Analysis

Outcomes assessed included body-weight recovery, DAI, histopathological damage, gene expression, IMC-derived tissue and cellular measures, and CyTOF immune-cell frequencies. As this was an exploratory study, no formal a priori sample-size calculation or single primary outcome measure was used to determine group size.

For comparisons between two independent groups, including IMC and CyTOF data analyses, normality was first assessed via the Shapiro–Wilk test. Normally distributed data were analyzed using an unpaired *t*-test, with Welch’s correction applied in instances of unequal variance as determined by an F-test. For non-normally distributed data, the non-parametric Mann–Whitney U test was employed.

As experiments involving three or more groups, DAI and histopathology analyses were first evaluated for normality and homogeneity of variance using the Shapiro–Wilk and Bartlett’s tests, respectively. Since assumptions of normality and equal variance were met, an ordinary one-way ANOVA followed by Dunnett’s post hoc test was performed for multiple comparisons against the control.

For gene expression analyses, data were transformed to fold-change values to evaluate the biological magnitude of treatment effects on wound healing related gene expression. Fold-change values were calculated using the 2^−ΔΔCt^ method, where target gene expression was normalized to the mean of internal housekeeping controls (*Actb* and *Gapdh*) and expressed relative to the untreated control group. Since control values were normalized to 1.0, the control group lacked variance by design, resulting in the technical violation of normality and homogeneity of variance (Shapiro–Wilk and Bartlett’s tests, respectively). Consequently, the non-parametric Kruskal–Wallis test followed by Dunn’s post hoc test was employed to determine significant differences between treatment groups and the normalized control. We note that with three animals per group this test has limited power, that rank-based methods are sensitive to small sample sizes, and that non-parametric testing does not compensate for a limited number of biological replicates. These results are therefore presented as exploratory.

For longitudinal data, such as daily body weight recovery, a two-way repeated measures ANOVA was employed to evaluate the main effects of treatment, time, and their interaction. To account for the observed non-uniform variance and violations of sphericity in both time and interaction factors (ε = 0.3400), the Geisser-Greenhouse correction was applied to adjust the degrees of freedom. This adjustment specifically addresses the non-uniform variance structure of the recovery period, where treatment-induced divergence in disease kinetics results in unequal variances across time points. Post hoc analysis was subsequently performed using Dunnett’s multiple comparisons test to identify significant deviations between treatment groups and the vehicle control at specific time points.

As this preclinical work is exploratory in scope, reported *p*-values were not adjusted for family-wise error rates, and as such, these data should be considered within the context of early-stage drug development and validated in future studies. All analyses were performed using GraphPad Prism (version 10.0.10, GraphPad Software, Boston, MA, USA).

## 3. Results

### 3.1. Acute OXA Colitis Induction

**Figure 1:**
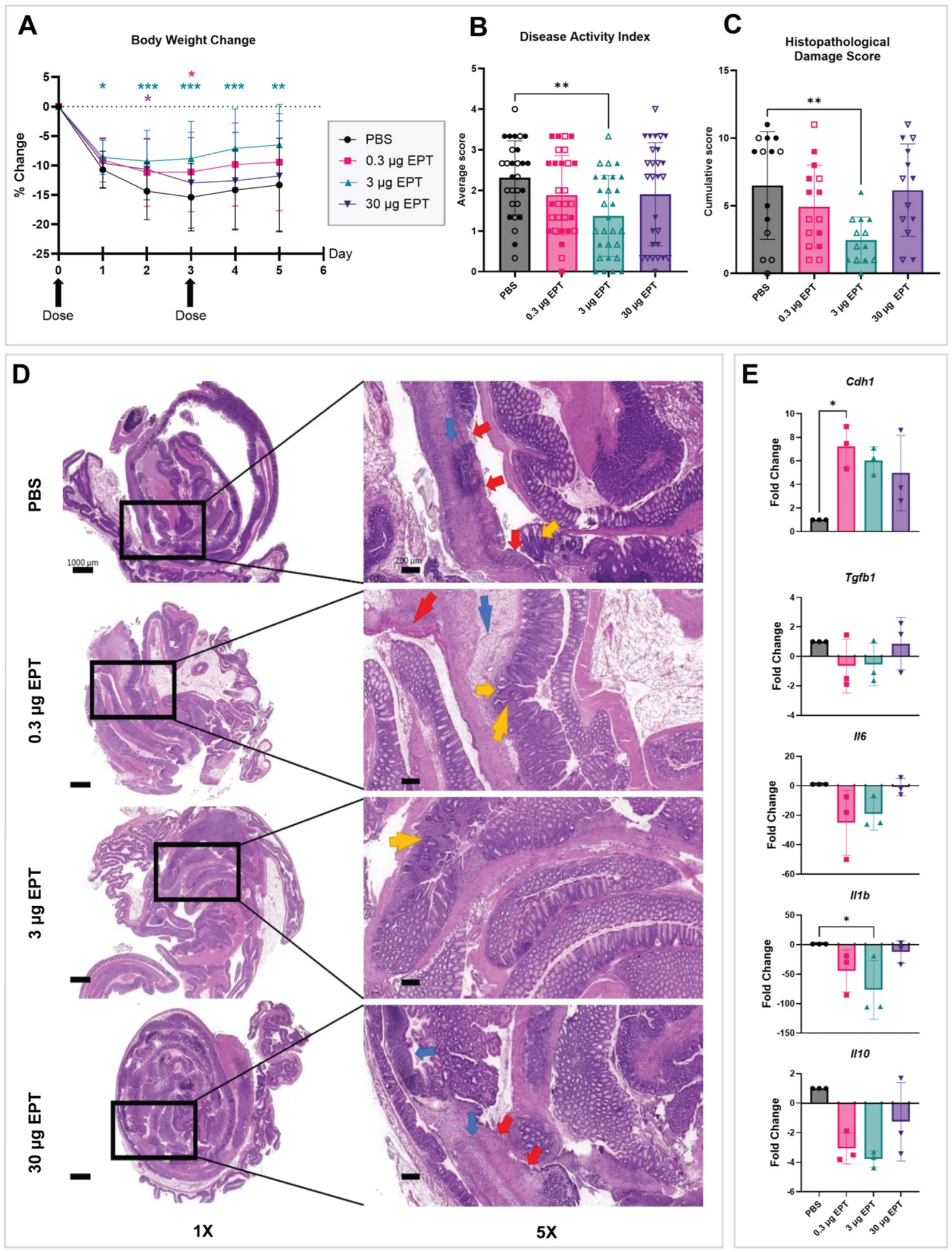
EPICERTIN ameliorates acute oxazolone (OXA) colitis. (**A**–**C**) Clinical and Histological Markers: Acute OXA colitis was induced in male and female BALB/c mice, and the efficacy of EPICERTIN (EPT) was evaluated by monitoring (**A**) body weight change, (**B**) disease activity index (DAI), and (**C**) histopathological damage scores of H&E-stained colon sections. Treatment with 3 µg EPT significantly mitigated all three disease markers, indicating a robust therapeutic effect. (**B**,**C**) Sex is indicated by the symbol fill: solid symbols represent males, and open (outline) symbols represent females. Data are presented as mean ± SD, with statistical significance determined by two-way repeated measures (**A**) or One-way (**B**,**C**) ANOVA with Dunnett’s multiple comparisons test (* *p* < 0.05, ** *p* < 0.01, *** *p* < 0.001). (**D**) Microscopic Amelioration of Colitis: Representative H&E-stained colon Swiss rolls of the male cohort at 1× (**left**) and 5× (**right**) magnification demonstrate the protective effect of EPT. The 3 µg EPT-treated group shows greater preservation of colonic architecture, with reduced inflammatory cell infiltration (small blue arrow), goblet cell depletion (small yellow arrow), ulceration (small red arrow), edema (large blue arrow), crypt loss (large yellow arrow), and muscular lesions (large red arrow) compared to other groups. Corresponding histopathological scores: PBS (9), 0.3 µg (6), 3 µg (2), and 30 µg (7). Scale bars are 1000 µm for 1× and 200 µm for 5× images. (**E**) Gene Expression Analysis: In the male cohort, qRT-PCR analysis of colon tissue reveals that EPT treatment significantly modulates the expression of genes associated with wound healing. Increased expression of *Cdh1* substantiates EPT-mediated mucosal repair via epithelial restitution. Data are presented as fold change relative to the PBS control (*n* = 3 per group). Since control values were normalized to 1.0, resulting in a lack of variance by design, statistical significance was determined by Kruskal–Wallis with Dunn’s multiple comparisons test (* *p* < 0.05). Given the number of targets and populations examined, *p*-values are reported without adjustment for family-wise error rates and therefore interpreted as exploratory.

The therapeutic efficacy of orally administered EPICERTIN was evaluated in a murine model of acute OXA colitis. Colitis development was evidenced by a significantly elevated Th2 population in the mesenteric lymph nodes (MLNs) of PBS-treated mice compared to healthy mice (Figure S1), as well as marked weight loss of approximately 15% body weight one day after challenge (Figure 1A). At the time of sacrifice, the PBS group displayed distinct histopathological damage including severe inflammatory cell infiltration across all layers of the colonic wall, sometimes involving the serosa and sometimes the peritoneum, goblet cell depletion, severe ulceration, edema, and muscular lesions (Figure 1D). A varied disease induction was observed in the PBS group, which may reflect a feature of the OXA-induced T cell-driven colitis model [23].

Across the main dose–response study, six animals were excluded because they did not meet the predefined criterion for successful colitis induction (<5% body-weight loss one day after OXA challenge): one male PBS-treated mouse, two male 30 µg EPICERTIN-treated mice, two female 3 µg EPICERTIN-treated mice, and one female 30 µg EPICERTIN-treated mouse. Three additional animals were found deceased following overnight monitoring intervals: one male in the 0.3 µg EPICERTIN group, one male in the 3 µg EPICERTIN group, and one female in the PBS group.

### 3.2. Efficacy of EPICERTIN in Acute OXA Colitis

To establish the optimal therapeutic range, we evaluated a broad, 10-fold dose range of EPICERTIN centered on 3 μg, a dose known to be efficacious in prior DSS colitis studies [14]. A two-way repeated measures ANOVA of body weight recovery revealed significant main effects of both time (F(1.700, 190.4) = 177.8, *p* < 0.0001) and treatment (F(3, 112) = 4.797, *p* = 0.0035). Additionally, a significant treatment × time interaction was observed (F(5.100, 190.4) = 4.000, *p* = 0.0017), which confirms that the therapeutic impact on weight gain diverged significantly over the study duration. To account for the observed violation of sphericity, all degrees of freedom were adjusted using the Geisser-Greenhouse correction (ε = 0.3400). Follow-up Dunnett-adjusted post hoc comparisons, identifying specific days when treatment impacts deviated significantly from the vehicle control, are detailed throughout the remainder of this section.

The 3 μg dose of EPICERTIN demonstrated the most robust amelioration of disease severity. This was evidenced by significantly enhanced and sustained body weight recovery starting at Day 1 (*p* = 0.0271), maintained through Day 5 (Days 2–4, *p* < 0.001; Day 5, *p* = 0.0027; Figure 1A). Consistent with this recovery, a significant reduction in the Disease Activity Index (DAI) was observed in the 3 μg EPICERTIN dose group compared to PBS control (Figure 1B). Histopathological evaluation confirmed these clinical findings, showing significantly reduced histopathological damage scores (Figure 1C), characterized by decreased inflammatory cell infiltration and preserved crypt architecture (Figure 1D; Table S1). The efficacy of the 3 μg dose is further corroborated by gene expression data. Treatment with 3 µg EPICERTIN significantly reduced *Il1b*, a pro-inflammatory cytokine; the 3 µg dose also demonstrated a trending upregulation of E-cadherin (*Cdh1*) (Figure 1E), which was previously observed in EPICERTIN treated human IBD explants and in DSS colitis [12]. Conversely, the 0.3 μg and 3 μg EPICERTIN groups unexpectedly showed a trending downregulation of the anti-inflammatory cytokine IL-10 at the gene level (Figure 1E), a finding not observed in prior DSS models. Expression of the complete assayed panel across all dose groups is shown in Figure S4.

Analysis of the remaining doses revealed a non-linear therapeutic response, which can occur with biological agents [25,26]. The 0.3 μg dose provided a significant but modest transient improvement in body weight recovery on Day 3 (*p* = 0.0314; Figure 1A) but did not significantly improve the DAI or histopathological damage score (Figure 1B,C). Notably, this dose was associated with a significant upregulation of *Cdh1* expression at the time of sacrifice (Figure 1E), a marker of late-stage epithelial repair and barrier homeostasis [27,28]. The 30 μg dose did not significantly improve or exacerbate the DAI or histopathological damage scores (Figure 1B,C), despite transiently improved body weight recovery at Day 2 (*p* = 0.0182; Figure 1A) and increased collagen gene expression (*Col1a2* and *Col3a1*; Figure S5) up to five-fold above the PBS control.

### 3.3. EPICERTIN Enhances Barrier Integrity and Controlled Tissue Repair

**Figure 2:**
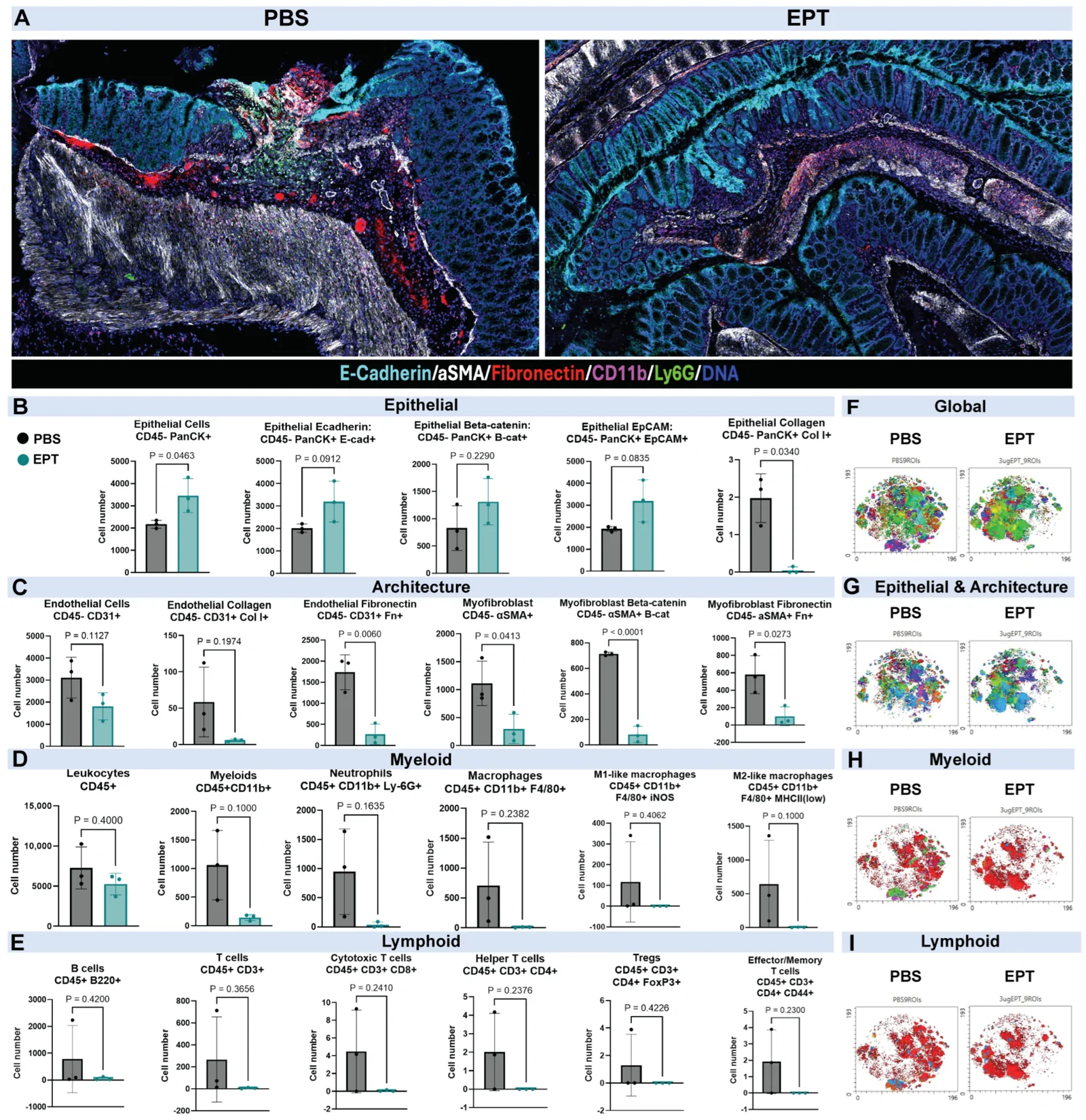
EPICERTIN-mediated mucosal healing via epithelial barrier restoration and resolution of local inflammatory cell infiltration. IMC analysis was performed on colonic tissue to visualize and quantify the effects of EPICERTIN (EPT). (**A**) Representative IMC images from a region of interest (ROI) per group (PBS and 3 µg EPT) illustrate the local expression of selected markers. Each ROI is 2.5 mm × 1.5 mm. (**B**) Epithelial panel: EPT treatment significantly increased the total number of pan-cytokeratin^+^ (PanCK) epithelial cells alongside trending increased expression of key epithelial barrier markers, including E-cadherin, β-catenin, and EpCAM, demonstrating an improved epithelial integrity. (**C**) Tissue architecture panel: EPT-treatment resulted in a significant beneficial reduction in markers associated with fibrotic response, such as activated myofibroblasts and endothelial cells expressing extracellular matrix proteins like collagen type I, β-catenin, and fibronectin. EPT was associated with a trending reduction in mucosal infiltration of inflammatory cells of both the myeloid (**D**) and lymphoid (**E**) lineages, including neutrophils and cytotoxic T cells of note. (**F**–**I**) Cell populations identified by IMC, displayed on a single shared t-SNE embedding computed from all concatenated samples, shown separately for the PBS and EPT groups. (**F**) All annotated populations. (**G**) CD45^−^ populations, comprising the epithelial and tissue architecture compartments. (**H**,**I**) CD45^+^ cells, with myeloid populations overlaid in (**H**) and lymphoid populations in (**I**); the two panels display the same CD45^+^ cells at the same coordinates and differ only in which populations are overlaid. Populations outside each panel’s scope are omitted from display. Panels are illustrative and show the spatial distribution of annotated populations; colors are assigned automatically by the analysis software and distinguish populations without encoding any quantitative property. Quantitative comparisons of population abundance are presented in (**B**–**E**). Data are presented as mean ± SD (*n* = 3 animals per group; 3 ROIs per animal, averaged within animals prior to analysis.); Statistical significance was determined using an unpaired *t*-test with Welch’s correction where appropriate, or Mann–Whitney test as a non-parametric equivalent. Given the number of targets and populations examined, *p*-values are reported without adjustment for family-wise error rates and therefore interpreted as exploratory.

IMC analysis provided deeper insights into the mechanisms by which EPICERTIN promotes tissue repair, revealing a dual mode of action: restoring the epithelial barrier and resolving inflammation. Representative IMC images from a ROI per group (PBS and 3 µg EPICERTIN) are shown (Figure 2A), illustrating the local expression of select markers (CD11b, Ly6G, E-cadherin, CD31, and DNA). For a complete overview, all analyzed ROIs can be found in Figure S6 and antibodies in Table S3. IMC analysis showed a trending increased expression of key epithelial markers, including E-cadherin, β-catenin, and epithelial cell adhesion marker (EpCAM) on pan-cytokeratin (PanCK)^+^ epithelial cells within the colon tissue upon treatment with EPICERTIN (Figure 2B), indicating a marked improvement in epithelial barrier integrity; further, EPICERTIN treatment significantly increased the total number of PanCK^+^ epithelial cells. These findings are consistent with the upregulation of *Cdh1* in the qPCR analysis (Figure 1E). Given the exploratory nature of this analysis and the number of targets and populations examined, *p*-values are reported without adjustment for family-wise error rates and should be validated in future studies. The IMC data also revealed a beneficial reduction in cell types and extracellular matrix (ECM) proteins associated with fibrotic response, including a trending decrease in endothelial cell numbers (CD45^−^ CD31^+^) and a significant decrease in myofibroblast numbers (CD45^−^ alpha-smooth muscle actin (α-SMA)^+^). Further, EPICERTIN significantly reduced myofibroblast expression of β-catenin and fibronectin alongside a significant reduction in endothelial-associated fibronectin (CD45^−^ CD31^+^ Fn^+^) and a trending decrease in endothelial collagen (CD45^−^ CD31^+^ Col I^+^) (Figure 2C). Treatment-associated changes were also reflected in t-SNE visualizations of the myeloid and lymphoid compartments (Figure 2H,I).

### 3.4. EPICERTIN Reduces Inflammatory Infiltration and Promotes a Healing Phenotype

EPICERTIN treatment was associated with reduced mucosal inflammatory infiltration (Figure 1C–E), a pro-healing effect confirmed by both IMC and CyTOF. The IMC analysis (Figure 2D,E) showed that EPICERTIN treatment reduced the local infiltration of both myeloid and lymphoid inflammatory cells, including neutrophils (CD45^+^ CD11b^+^ Ly6G^+^) and cytotoxic T cells (CD45^+^ CD3^+^ CD8^+^). While these differences did not reach statistical significance at the animal level, these cell types are central to inflammation propagation and epithelial damage in UC [29]. Treatment-associated changes were also reflected in t-SNE visualizations of the myeloid and lymphoid compartments (Figure 2H,I).

**Figure 3:**
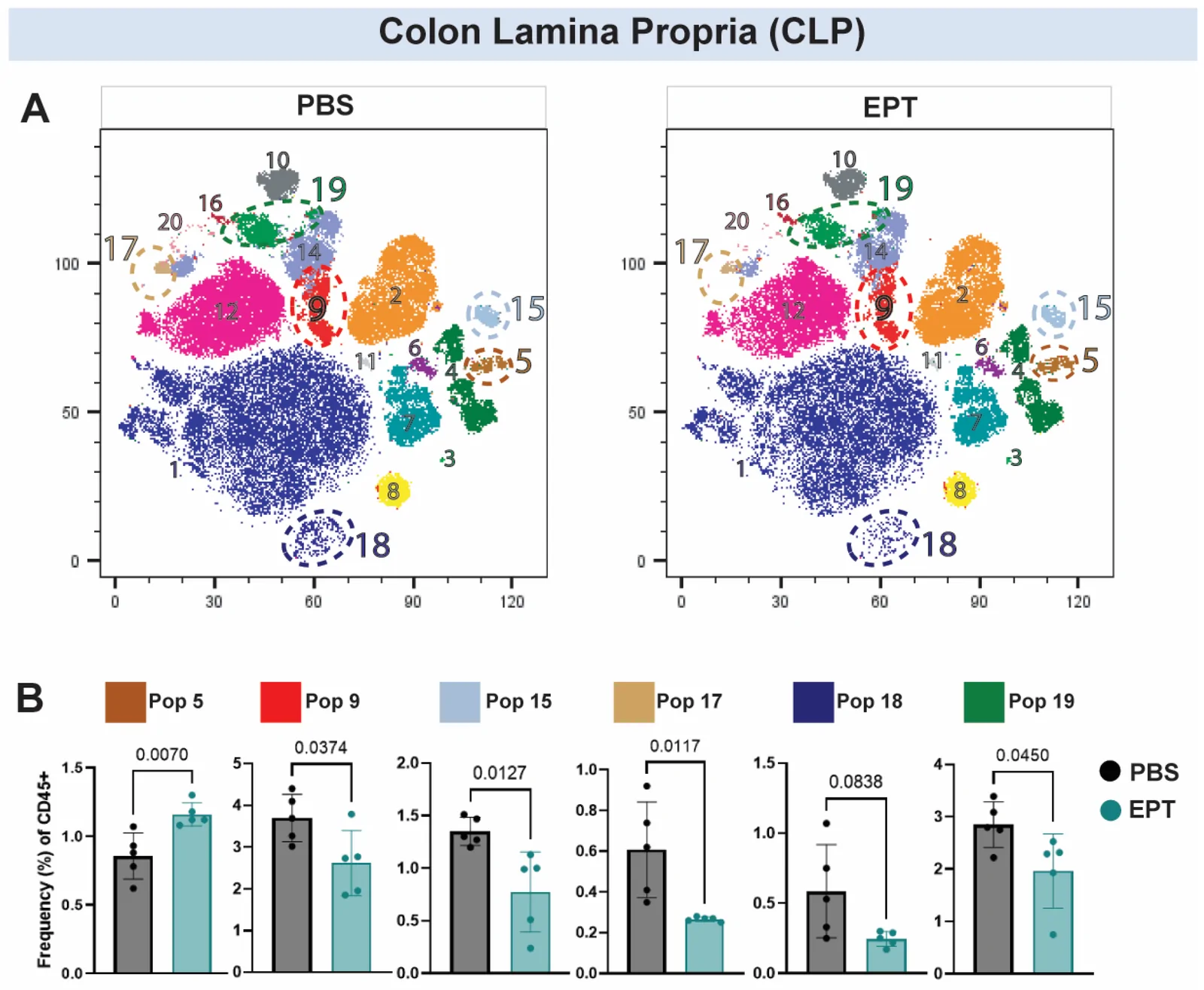
EPICERTIN favorably modulates immune cell populations in the colon lamina propria (CLP) during acute OXA colitis. CyTOF analysis of EPICERTIN (EPT) treatment in acute OXA colitis. (**A**) t-SNE plots of CLP single-cell distributions annotated by FlowSOM clusters. Selected populations corresponding to quantitative analyses in (**B**) are outlined with dashed lines. The t-SNE plots are annotated with all FlowSOM populations except Pop 13, which was not sufficiently abundant for reliable annotation. The corresponding population colors are retained and explicitly identified in (**B**) to facilitate comparison across panels; these colors are categorical and do not encode population abundance, effect size, or statistical significance. (**B**) Relative abundance of key CLP immune populations expressed as frequency (%) of CD45^+^ cells. Major FlowSOM clusters were annotated according to dominant marker expression: Pop 5 = γδ T cells; Pop 9 = type 2 conventional dendritic cells (cDC2s); Pop 15 = unclassified antigen presenting cells; Pop 17 = newly tissue-infiltrating inflammatory monocytes; Pop 18 = neutrophils; and Pop 19 = anti-inflammatory tissue-resident macrophages. EPT treatment reduced the frequencies of several myeloid populations (Pops 9, 15, 17, and 19) while increasing γδ T cells (Pop 5). Data are presented as mean ± SD (*n* = 5 mice/group). Statistical significance was determined using an unpaired *t*-test (with Welch’s correction where appropriate) or the Mann–Whitney test. Given the number of targets and populations examined, *p*-values are reported without adjustment for family-wise error rates and therefore interpreted as exploratory.

CyTOF analysis of the CLP (Figure 3A,B) further demonstrated that EPICERTIN broadly reduced inflammatory cell populations, reshaping immune populations in the inflamed colon. In the CLP (Figure 3A,B) EPICERTIN significantly reduced the frequencies of type 2 conventional dendritic cells (cDC2s; Pop 9), unclassified antigen presenting cells (Pop 15), newly tissue-infiltrating inflammatory monocytes (Pop 17), and anti-inflammatory tissue-resident macrophages (Pop 19), while increasing γδ T cells (Pop 5) (Figure 3A,B). Although neutrophils (Pop 18) were not significantly changed, their frequency trended lower following EPICERTIN treatment. Notably, Pop 19 expressed arginase 1 and was annotated as a macrophage population with a putative reparative phenotype; because CyTOF data are reported as relative frequencies of CD45^+^ cells at a single time point, the observed reduction may reflect altered macrophage recruitment, differentiation, or tissue-resident macrophage dynamics. CyTOF antibody information is provided in Supplementary Table S4, and FlowSOM cluster annotations based on canonical marker expression are detailed in Supplementary Table S5.

Together, these findings demonstrate that EPICERTIN attenuates mucosal inflammation and reshapes immune cell composition toward a tissue-protective phenotype that supports epithelial repair.

## 4. Discussion

The present study demonstrates that the oral administration of *E. coli*-derived EPICERTIN effectively ameliorates acute OXA-induced colitis by facilitating epithelial barrier restoration and modulating the adaptive immune milieu. The identification of 3 μg as the optimal therapeutic dose is consistent with previous findings in DSS models, thereby reinforcing the therapeutic window across disparate pathophysiological environments. The observed non-linear dose response, characterized by diminished efficacy at 30 μg (Figure 1B,C), may reflect a recognized phenomenon in biotherapeutic development [25,26]. Although the mechanism underlying the lack of therapeutic benefit at this highest dose remains unclear, it may involve overstimulation of the UPR or KDELR-mediated signaling. Our previous studies in mice and human IBD colonic explants demonstrated that EPICERTIN promotes epithelial repair through upregulation of *TGFβ1*, *WNT5A*, and *CDH1* [12], among other wound-healing related genes. We therefore speculate that a high dose may disrupt the balance of TGFβ and canonical/noncanonical Wnt-signaling pathways necessary for enhanced epithelial repair reflected by increased E-cadherin expression (Figure 1E and Figure 2B). This observation is consistent with previous DSS colitis studies in which a 30 μg dose did not confer superior efficacy [14]. Importantly, we found no histopathological evidence of adverse effects with the 30 μg dose, consistent with our prior findings in healthy and DSS colitis mice [14], suggesting a promising safety profile for EPICERTIN. Furthermore, no significant changes in gross mortality or population loss due to humane endpoints occurred during the course of the study. Given the clear efficacy observed with the 3 μg dose, the optimal therapeutic range likely lies between 0.3 μg and 3 μg, providing a foundation for future studies focusing on further refining dosing strategies. Overall, these results demonstrate robust amelioration of acute OXA colitis via oral administration of EPICERTIN dosed twice at 3 μg.

Our previous studies have shown that EPICERTIN activates TGFβ1-associated wound healing pathways [12,14]. TGFβ1 signaling is a key regulator of intestinal wound repair [30,31] and has also been implicated in regenerative responses associated with revival stem cells (revSCs) [31,32,33]. Although revSC markers were not directly evaluated in the current study, the observed increases in epithelial integrity markers (Figure 2B), together with reduced inflammatory cell infiltration (Figure 2D,E and Figure 3), are consistent with activation of coordinated regenerative responses that promote restoration of epithelial homeostasis. Future studies investigating the effects of EPICERTIN on revSC induction and fetal-like epithelial reprograming will be important to further define the cellular mechanisms underlying its mucosal healing activity.

An important consideration for therapies that promote TGFβ1-associated tissue repair is the potential for excessive ECM deposition and fibrosis resulting from dysregulated or sustained TGFβ1 signaling [34,35]; however, our findings suggest EPICERTIN promotes a balanced repair process without evidence of pathological fibrotic remodeling. This conclusion is supported by the preservation of epithelial integrity, reflected by increased trending expression of epithelial markers (PanCK, E-cadherin, and EpCAM), together with reduced expression of remodeling-associated markers (collagen type I, α-SMA, and fibronectin; Figure 2B,C). Consistent with these observations, EPICERTIN treatment did not increase the expression of *Tgfb1* or *Il6* at the time of sacrifice (Figure 1E). While mechanistic interpretation is limited with the present study design, these findings suggest that EPICERTIN facilitates physiological mucosal repair while limiting pathological tissue remodeling, although studies in chronic models are needed to evaluate its long-term effects on fibrosis. Notably, Th2 inflammation, a hallmark of the OXA model, has been implicated in fibrotic complications in human IBD [36]. The absence of profibrotic changes despite treatment in this model therefore further supports the safety of EPICERTIN in a fibrogenic inflammatory environment. Consistent with the present findings, our previous study demonstrated that pretreatment with 30 μg of EPICERTIN in DSS mice reduced the expression of fibrosis-associated genes (*Col1a1* and *Tgfb1*) and decreased collagen deposition, as assessed by Masson’s trichrome staining [14]. Collectively, these results indicate that EPICERTIN promotes regenerative mucosal healing while avoiding excessive fibrotic remodeling in acute experimental colitis.

IMC and CyTOF analyses identified immune changes that may link EPICERTIN-mediated epithelial restitution with resolution of inflammation. In the CLP, EPICERTIN reduced the frequencies of several myeloid populations, including cDC2s, unclassified antigen presenting cells, newly tissue-infiltrating inflammatory monocytes, and a macrophage cluster with a putative reparative phenotype, while increasing γδ T cells (Figure 2D,E and Figure 3A,B). The increased γδ T-cell frequency may contribute to the tissue-protective profile observed following EPICERTIN treatment, given their established roles in epithelial surveillance, inflammation resolution, and tissue repair [3,37]. However, because these analyses reflect relative cell frequencies at a single time point, functional and longitudinal studies in a chronic colitis model will be needed in the future to define the contribution of individual immune populations to therapeutic response.

Several limitations and future directions should be borne in mind. Oxazolone-induced colitis reproduces a number of the Th2-associated immunological and histopathological features of human UC and is well suited to the study of acute epithelial injury and repair, but it remains an acute, hapten-induced model driven by a defined chemical insult, and it does not recapitulate the chronic, relapsing–remitting course, the microbial contribution, or the genetic heterogeneity of human disease. Since animals were euthanized shortly after disease induction, the present study cannot address whether the epithelial repair observed is durable, nor whether the increased collagen gene expression seen at the highest dose would progress to established fibrosis over a longer period. Determining this will require chronic models with extended follow-up and direct histological and biochemical assessment of fibrosis. IMC was performed on three animals per group; this limited number of biological replicates constrains statistical power, and the immune population differences observed should be regarded as exploratory. Finally, the high-dimensional analyses were conducted in male animals only, so their generalizability to female animals remains to be established. Findings obtained in this model should therefore be regarded as supporting continued preclinical evaluation rather than as directly predictive of clinical response.

The next stage of development will involve evaluation in chronic and translationally relevant models with extended follow-up, beginning with the chronic oxazolone model, together with formal dose-ranging studies to define the therapeutic window suggested by the non-linear response observed here. Estimation of a human dose range will need to account for the topical, luminal mode of action of an orally delivered protein, and local concentration at the colonic mucosal surface. Candidate pharmacodynamic and efficacy measures for early clinical evaluation may likely include endoscopic and histological indices of mucosal healing, epithelial repair-associated transcripts in mucosal biopsies, and established non-invasive markers of intestinal inflammation such as fecal calprotectin.

Of note, EPICERTIN is derived from the B subunit of cholera toxin which does not contain the enzymatically active A subunit responsible for the secretory diarrhea characteristic of cholera. Recombinant CTB has an extensive record of human administration, including as a component of licensed oral cholera vaccines, and is generally well tolerated by the oral route [38]. Nonetheless, the potential for immunogenicity, including the development of anti-CTB antibodies and any consequent loss of activity or hypersensitivity with repeated administration, will require systematic evaluation, as will local mucosal tolerance and the effects of chronic dosing on epithelial turnover. These questions will be addressed in the formal toxicology program preceding any clinical evaluation.

## 5. Conclusions

In conclusion, these results demonstrate that EPICERTIN, orally administered twice at 3 μg, exhibits robust therapeutic efficacy in the acute OXA colitis mouse model. EPICERTIN’s demonstrated epithelial restitution in the OXA model represents a meaningful step in its preclinical development for the treatment of UC. Integrated with prior evidence from human IBD colon explants and DSS colitis models, these findings support further preclinical development and evaluation of EPICERTIN in additional chronic and translationally relevant models of ulcerative colitis.

## Data Availability

The original contributions presented in this study are included in the article/Supplementary Materials. Further inquiries can be directed to the corresponding author.

## Supplementary Materials

The following supporting information can be downloaded at https://www.mdpi.com/article/10.3390/biomedicines14081777/s1. Figure S1: Significant expansion of Th2 cells in mesenteric lymph nodes (MLNs) during acute oxazolone-induced colitis; Figure S2: Sex-stratified body weight recovery in male and female mice; Figure S3: Sex-stratified disease activity index and histopathological damage scores; Table S1: Histopathological damage scoring parameters and corresponding raw data; Figure S4. Complete RT-qPCR target heatmap panel across all dose groups; Figure S5: Upregulation of collagen gene expression in EPICERTIN-treated groups; Figure S6: All imaging mass cytometry (IMC) regions of interest (ROIs); Table S2: RT-qPCR primer information; Table S3: IMC antibody panel; Table S4: Cytometry by Time of Flight (CyTOF) antibody panel; Table S5: Putative FlowSOM population annotations for CyTOF analysis of colon lamina propria (CLP).

## Abbreviations

The following abbreviations are used in this manuscript:

IBD	Inflammatory bowel disease
UC	Ulcerative colitis
CD	Crohn’s disease
GI	Gastrointestinal
FDA	Food and Drug Administration
CTB	Cholera toxin B subunit
ER	Endoplasmic reticulum
EPICERTIN (EPT)	Epithelial Cell ER-Targeted Protein
UPR	Unfolded protein response
KDELR	KDEL receptor
DSS	Dextran sulfate sodium
OXA	Oxazolone
PanCK	pan-cytokeratin
EpCAM	epithelial cell adhesion marker
α -SMA	alpha-smooth muscle actin
IMAC	Immobilized metal affinity chromatography
CHT	Ceramic hydroxyapatite
TB	Terrific Broth
PBS	Phosphate-buffered saline
SEC-HPLC	Size exclusion chromatography high-performance liquid chromatography
UCAW	University Committee for Animal Welfare
IACUC	Institutional Animal Care and Use Committee
DAI	Disease Activity Index
H&E	Hematoxylin and eosin
qRT-PCR	Quantitative reverse transcription polymerase chain reaction
cDNA	complementary DNA
*Cdh1*	Cadherin 1 (E-cadherin)
*Tgfb1*	Transforming growth factor beta 1
*Wnt5a*	Wnt family member 5A
*Col1a2*	Collagen type I alpha 2 chain
*Col3a1*	Collagen type III alpha 1 chain
*Col4a1*	Collagen type IV alpha 1 chain
*Mmp2*	Matrix metallopeptidase 2
*Ctsk*	Cathepsin K
*Ctsg*	Cathepsin G
*Csf3*	Colony stimulating factor 3 (G-CSF)
*Tagln*	Transgelin
*Angpt1*	Angiopoietin 1
*Il6*	Interleukin 6
*Il10*	Interleukin 10
*Il1b*	Interleukin 1 beta
*Cxcl3*	C-X-C motif chemokine ligand 3
*Ccl7*	C-C motif chemokine ligand 7
*Serpine1*	Serpin family E member 1 (PAI-1)
*Ifng*	Interferon gamma
*Actb*	Actin beta
*Gapdh*	Glyceraldehyde-3-phosphate dehydrogenase
Ct	Cycle threshold
IMC	Imaging Mass Cytometry
ROIs	Regions of interest
CLP	Colon lamina propria
MLNs	Mesenteric lymph nodes
CyTOF	Cytometry by Time of Flight
FlowSOM	Flow self-organizing map
t-SNE	t-distributed stochastic neighbor embedding
ECM	Extracellular matrix
Th	T helper
Treg	T regulatory
γδ T	Gamma delta T Cell
