# Supplementary material for: Amelioration of Acute Oxazolone-Induced Colitis via Oral Administration of EPICERTIN, a Mucosal Healing Biotherapeutic for Inflammatory Bowel Disease
Source: Biomedicines. 2026 Aug 6;14(8):1777. doi: 10.3390/biomedicines14081777 (PMC13509488; doi:10.3390/biomedicines14081777)
Supplement: Supplementary file 1 [file biomedicines-14-01777-s001.zip › biomedicines-4445448-supplementary.pdf]

Supplementary Materials

Amelioration of Acute Oxazolone-induced Colitis via Oral Administration of EPICERTIN, a Mucosal Healing Biotherapeutic for Inflammatory Bowel Disease

Wendy M. Kittle [1], Micaela A. Henderson [1], Noel Verjan Garcia [2], Hong Li [3], Katarina L. Mayer [1], Jimmy F. Cifuentes Jimenez [1], Kelly M. Lee [4], Kavitha Yaddanapudi [3,5], and Nobuyuki Matoba [1,2,3,*]

1 Department of Pharmacology and Toxicology, School of Medicine, University of Louisville, Louisville, KY 40292, USA; wendy_kittle@outlook.com (W.M.K.); hendersonm@hanover.edu (M.A.H.); katarina.mayer@louisville.edu (K.L.M.); jimmy.cifuentes@louisville.edu (J.F.C.J.)
2 Center for Predictive Medicine, School of Medicine, University of Louisville, Louisville, KY 40202, USA; nverjan@gmail.com
3 Brown Cancer Center, School of Medicine, University of Louisville, Louisville, KY 40202, USA; hong.li@louisville.edu (H.L.); kavitha.yaddanapudi@louisville.edu (K.Y.)
4 Department of Chemical Engineering, JB Speed School of Engineering, University of Louisville, Louisville, KY 40292, USA; kelly.lee.1@louisville.edu
5 Department of Surgery, School of Medicine, University of Louisville, Louisville, KY 40202, USA
* Correspondence: n.matoba@louisville.edu; Tel.: +1-502-852-8412

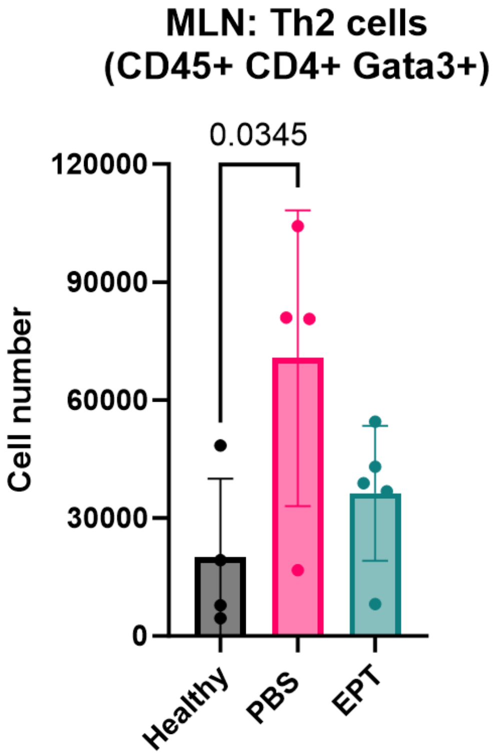

Figure S1. Significant expansion of Th2 cells in mesenteric lymph nodes (MLNs) during acute oxazolone-induced colitis.

In an independent, complementary study, male BALB/c mice with oxazolone-induced colitis were treated with PBS (n=4) or EPICERTIN (EPT) (3 µg, n=5) on Days 0 and 3, with an age-matched healthy control group (n=4) for comparison. Flow cytometric analysis of MLNs shows that oxazolone-induced colitis leads to a significant expansion of the Th2 cell population (CD45+ CD4+ Gata3+) in the PBS-treated group relative to healthy control. Data represent an initial cohort of n=5 per treatment group and n=4 healthy controls; final analyzed group sizes are Healthy (n=4), PBS (n=4), and EPT (n=5) following predefined exclusion criteria. Statistical significance was determined by one-way ANOVA with Dunnett's post-hoc correction (p=0.0345). No significant differences were observed in other T cell subsets within the MLNs relative to healthy controls.

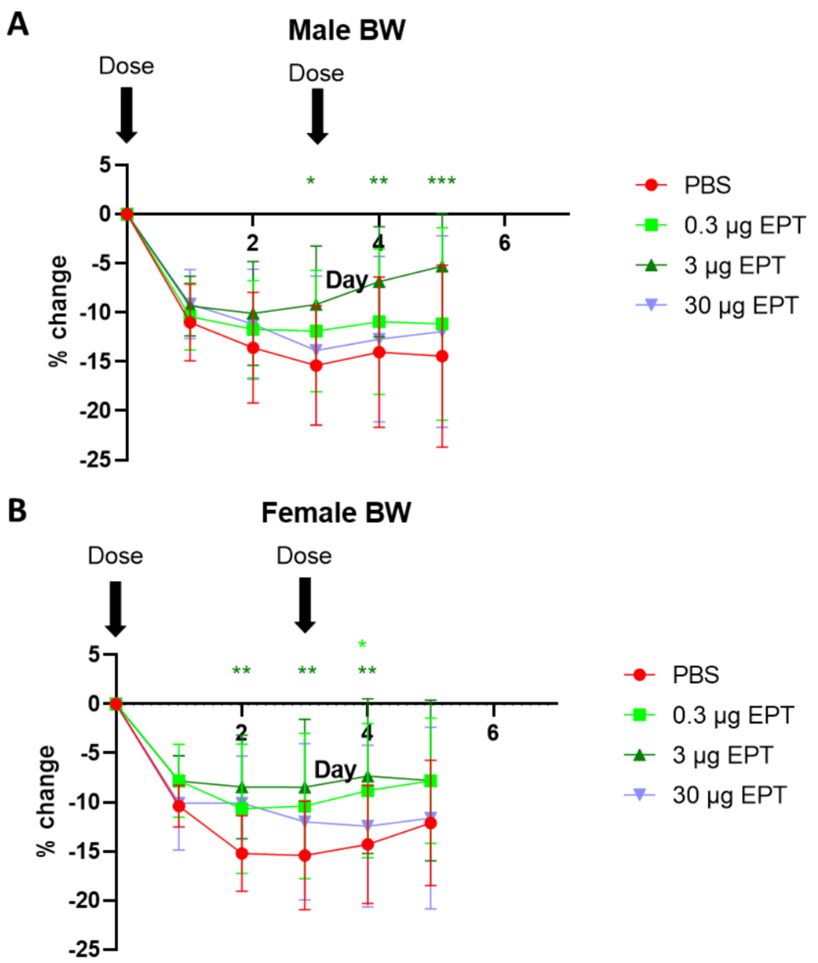

Figure S2. Sex-stratified body weight recovery in male and female mice.

Male and female mice with oxazolone (OXA)-induced colitis were treated with PBS or EPICERTIN (EPT; 0.3, 3, or 30 µg) on Days 0 and 3, as indicated by arrows. Percent body weight change over time is shown for (A) male and (B) female mice. Male mice treated with 3 µg EPT showed the greatest body weight recovery. Female mice treated with 0.3 or 3 µg EPT showed less body weight loss and faster recovery, although differences were not significant at the time of euthanasia as the PBS group had also begun to recover. Statistical significance was determined by two-way ANOVA with Dunnett's multiple comparisons test; *$P < 0.05$, **$P < 0.01$, ***$P < 0.0001$.

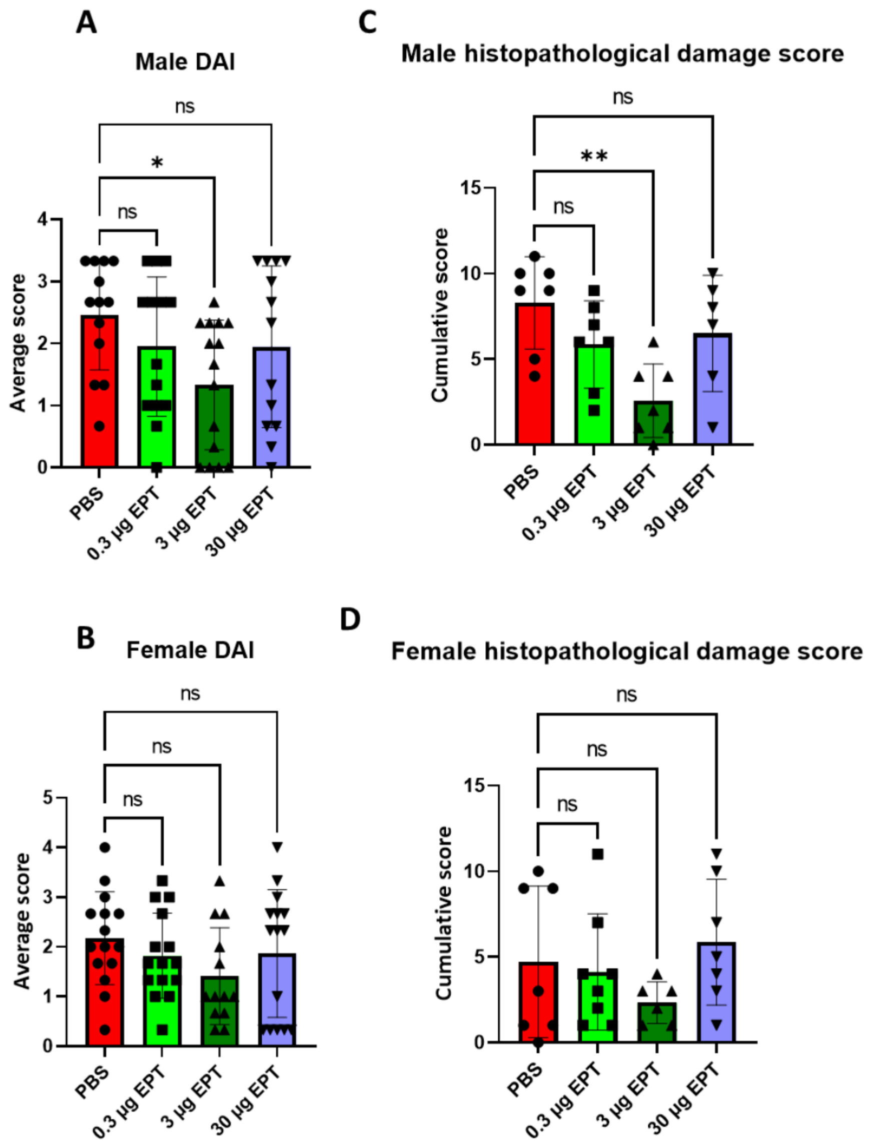

Figure S3. Sex-stratified disease activity index (DAI) and histopathological damage scores.

DAI scores at the time of euthanasia are shown for (A) male and (B) female mice. Histopathological damage scores for H&E-stained, Swiss-rolled whole colons are shown for (C) male and (D) female mice. DAI scores had a maximum of 3.33 for mice completing the study and 4 for mice euthanized before study completion. Histopathological damage was scored based on inflammatory cell infiltration, reduction of crypts/goblet cells, epithelial erosion/ulceration, and epithelial hyperplasia/crypt structure alterations, yielding a maximum cumulative score of 12. In male mice, 3 µg EPICERTIN (EPT) significantly reduced (A) DAI scores and (C) histopathological damage scores relative to PBS. No significant treatment effects were observed in female mice for (B) DAI or (D) histopathological damage scores. Data are presented as mean ± SD. Statistical significance was determined by one-way ANOVA with Dunnett's multiple comparisons test; *$P < 0.05$, **$P < 0.01$; ns, not significant.

Table S1. Histopathological damage scoring parameters and corresponding raw data.

		Scoring Parameters				
		Inflammatory cell infiltration	Reduction of crypts/goblet cells	Erosions/ Ulceration	Epithelial hyperplasia/ crypt structure alterations	Score Total/Sum (Max 12)
		None (0)	None (0)	None (0)	None (0)	
		Mild (1)	Mild (1)	Mild (1)	Mild (1)	
		Moderate (2)	Moderate (2)	Moderate (2)	Moderate (2)	
		Severe (3)	Severe (3)	Severe (3)	Severe (3)	
Experimental Damage Scores						
Treatment	Sex/Mouse ID#	Inflammatory cell infiltration	Reduction of crypts/goblet cells	Erosions/ Ulceration	Epithelial hyperplasia/ crypt structure alterations	Score Total/Sum (Max 12)
PBS	M1	3	1	0	0	4
PBS	M2	3	3	3	2	11
PBS	M3	3	2	2	3	10
PBS	M7	2	1	0	2	5
PBS	M10	3	3	3	0	9
PBS	M11	3	2	3	2	10
PBS	M12	3	3	1	2	9
0.3 μg EPT	M16	0	0	1	1	2
0.3 μg EPT	M17	2	2	0	2	6
0.3 μg EPT	M18	3	1	1	2	7
0.3 μg EPT	M21	3	0	0	0	3
0.3 μg EPT	M22	3	3	3	0	9
0.3 μg EPT	M26	2	1	0	3	6
0.3 μg EPT	M27	3	1	2	2	8
3 μg EPT	M31	3	1	0	0	4
3 μg EPT	M32	3	1	0	2	6
3 μg EPT	M33	0	0	0	0	0
3 μg EPT	M36	0	0	0	1	1
3 μg EPT	M37	0	2	0	0	2
3 μg EPT	M41	3	0	0	1	4
3 μg EPT	M42	1	0	0	0	1
30 μg EPT	M46	3	2	2	1	8
30 μg EPT	M47	3	1	1	2	7
30 μg EPT	M48	3	3	3	1	10
30 μg EPT	M52	1	1	0	2	4
30 μg EPT	M56	3	3	0	3	9
30 μg EPT	M57	1	0	0	0	1
PBS	F1	1	0	0	0	1
PBS	F2	3	2	3	2	10

PBS	F3	3	2	3	1	9
PBS	F6	0	0	0	0	0
PBS	F10	0	0	1	0	1
PBS	F11	0	1	0	2	3
PBS	F12	3	2	3	1	9
0.3 μg EPT	F16	3	0	0	1	4
0.3 μg EPT	F17	2	0	1	0	3
0.3 μg EPT	F18	0	0	0	1	1
0.3 μg EPT	F21	3	3	3	2	11
0.3 μg EPT	F22	1	0	1	0	2
0.3 μg EPT	F23	0	0	1	0	1
0.3 μg EPT	F26	2	1	0	1	4
0.3 μg EPT	F27	3	2	1	1	7
3 μg EPT	F31	1	0	0	0	1
3 μg EPT	F32	1	0	0	1	2
3 μg EPT	F33	2	0	0	1	3
3 μg EPT	F36	1	1	0	1	3
3 μg EPT	F41	3	0	0	1	4
3 μg EPT	F42	1	0	0	0	1
30 μg EPT	F46	2	0	1	1	4
30 μg EPT	F47	1	0	1	1	3
30 μg EPT	F48	3	3	3	2	11
30 μg EPT	F51	0	0	1	0	1
30 μg EPT	F52	3	3	3	1	10
30 μg EPT	F56	2	2	2	1	7
30 μg EPT	F57	2	1	1	1	5

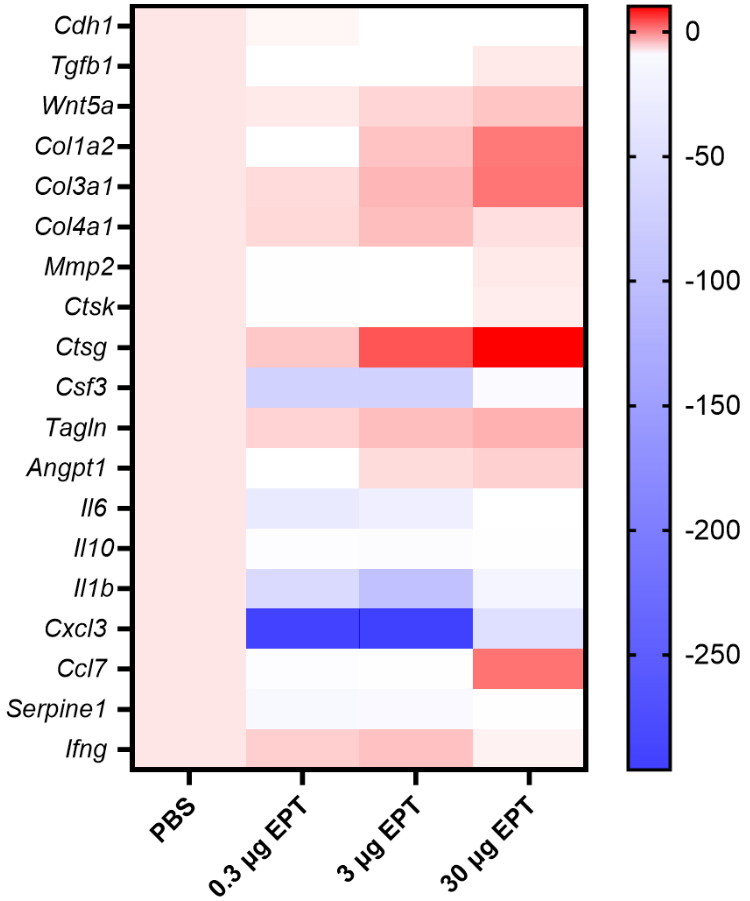

Figure S4. Complete RT-qPCR target heatmap panel across all dose groups.

Expression of all 19 assayed targets in distal colon tissue from mice treated with PBS, 0.3 µg, 3 µg or 30 µg EPICERTIN (EPT). Values are group means (n = 3 per group) expressed as fold regulation relative to the PBS control, calculated by the $2^{-\Delta\Delta Ct}$ method with normalization to *Actb* and *Gapdh*. Fold regulation is reported as fold change for values ≥ 1 and as the negative reciprocal of fold change for values < 1, such that positive and negative values denote increased and decreased expression, respectively. Since control values were normalized to 1.0, the PBS group lacks variance by design. Individual data points and statistical comparisons for selected targets are shown in Figure 1E; collagen gene expression is shown in Figure S5, and assay details for all targets are provided in Supplementary Table S2. Given the number of targets and populations examined, p-values are reported without adjustment for family-wise error rates and therefore interpreted as exploratory.

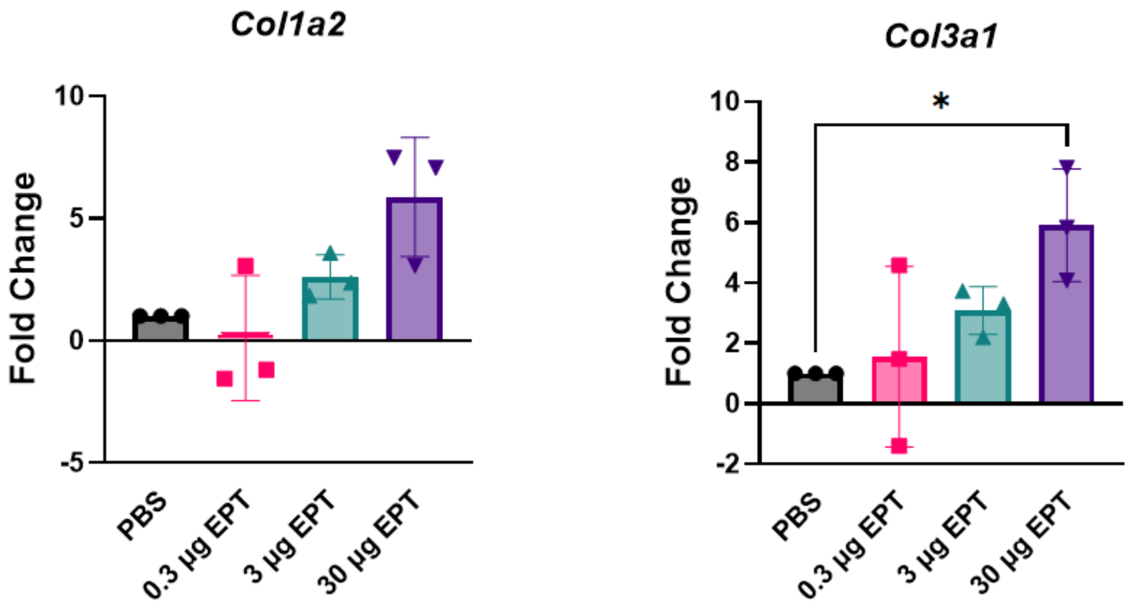

Figure S5. Upregulation of collagen gene expression in EPICERTIN-treated groups. Quantitative qRT-PCR analysis of colon tissue was evaluated in the male cohort. Data are presented as fold change relative to the PBS control (n = 3 per group). Since control values were normalized to 1.0, resulting in a lack of variance by design, statistical significance was determined by Kruskal Wallis with Dunn's multiple comparisons test (*$p < 0.05$).

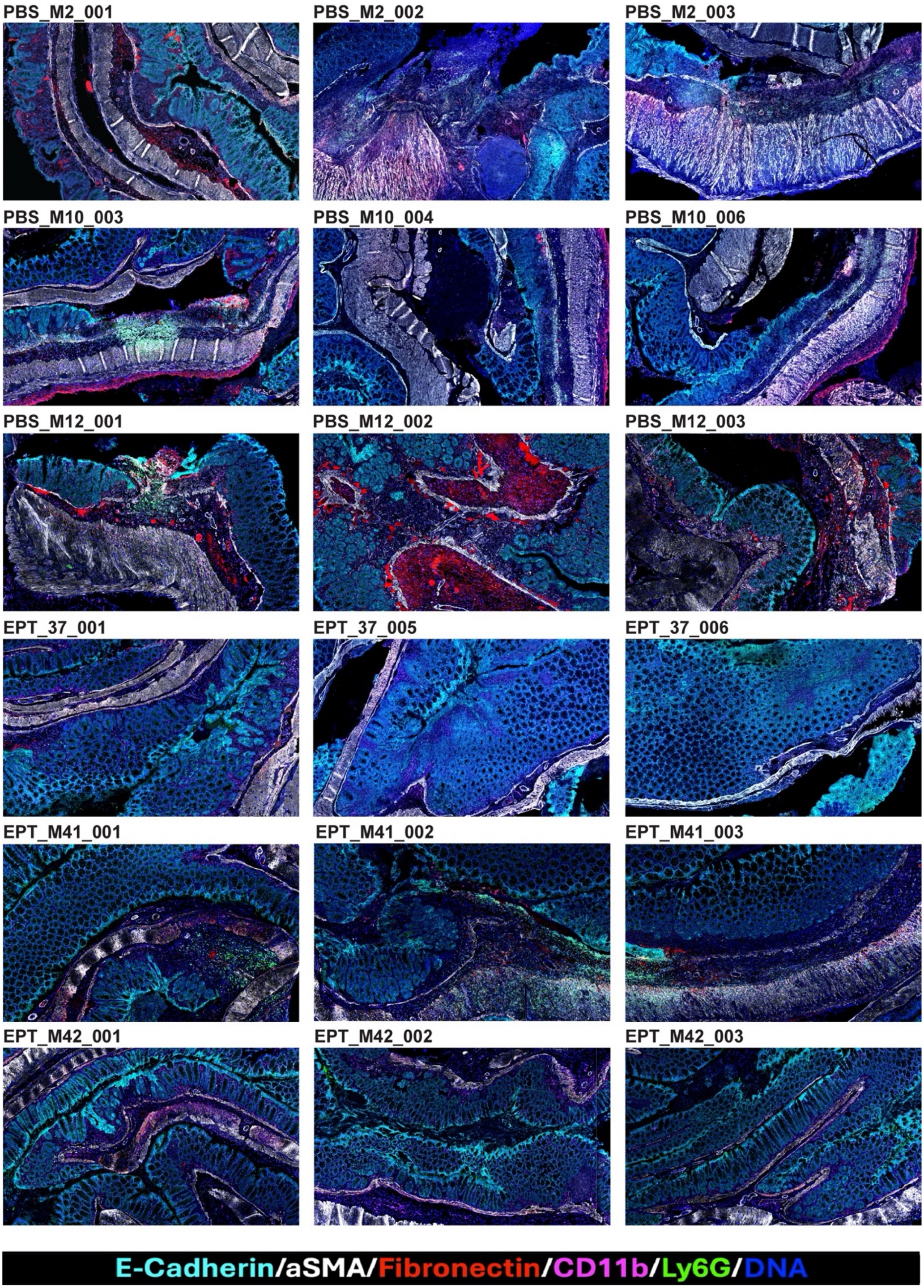
PBS_M2_001
PBS_M2_002
PBS_M2_003
PBS_M10_003
PBS_M10_004
PBS_M10_006
PBS_M12_001
PBS_M12_002
PBS_M12_003
EPT_37_001
EPT_37_005
EPT_37_006
EPT_M41_001
EPT_M41_002
EPT_M41_003
EPT_M42_001
EPT_M42_002
EPT_M42_003
E-Cadherin/aSMA/Fibronectin/CD11b/Ly6G/DNA

Figure S6. All imaging mass cytometry (IMC) regions of interest (ROIs).

All 2.5 mm × 1.5 mm ROIs selected for IMC analysis are shown, illustrating the total colon tissue area analyzed across samples. A representative marker panel is displayed: E-cadherin (cyan), α-SMA (white), fibronectin (red), CD11b (magenta), Ly6G (green), and DNA (blue). The top half shows the nine ROIs from the PBS group, and the bottom half shows the nine ROIs from the EPICERTIN (EPT) group.

Table S2. RT-qPCR primer information.

Gene symbol	Gene name	RefSeq ID	Entrez ID	Assay ID	Amplicon (bp)	Role
Cdh1	cadherin 1	NM_009864	12550	PPM03652F	147	Target
Tgfb1	transforming growth factor, beta 1	NM_011577	21803	PPM02991B	63	Target
Wnt5a	wingless-type MMTV integration site family, member 5A	NM_009524	22418	PPM04748C	85	Target
Col1a2	collagen, type I, alpha 2	NM_007743	12843	PPM04448F	64	Target
Col3a1	collagen, type III, alpha 1	NM_009930	12825	PPM04784B	173	Target
Col4a1	collagen, type IV, alpha 1	NM_009931	12826	PPM05145A	109	Target
Mmp2	matrix metallopeptidase 2	NM_008610	17390	PPM03642C	63	Target
Ctsk	cathepsin K	NM_007802	13038	PPM05123C	63	Target
Ctsg	cathepsin G	NM_007800	13035	PPM03849A	124	Target
Csf3	colony stimulating factor 3 (granulocyte)	NM_009971	12985	PPM02989B	80	Target
Tagln	transgelin	NM_011526	21345	PPM37374A	115	Target
Angpt1	angiopoietin 1	NM_009640	11600	PPM03054F	77	Target
Il6	interleukin 6	NM_001314054	16193	PPM03015A	178	Target
Il10	interleukin 10	NM_010548	16153	PPM03017C	81	Target
Il1b	interleukin 1 beta	NM_008361	16176	PPM03109F	119	Target
Cxcl3	chemokine (C-X-C motif) ligand 3	NM_203320	330122	PPM34590C	74	Target
Ccl7	chemokine (C-C motif) ligand 7	NM_013654	20306	PPM02955B	99	Target
Serpine1	serine (or cysteine) peptidase inhibitor, clade E, member 1	NM_008871	18787	PPM03093C	95	Target
Ifng	interferon gamma	NM_008337	15978	PPM03121A	95	Target
Actb	actin, beta	NM_007393	11461	PPM02945B	154	Reference
Gapdh	glyceraldehyde-3-phosphate dehydrogenase	NM_008084	14433	PPM02946E	140	Reference

Table S3. IMC antibody panel.

#	Label	Target (mouse)	Clone	Vendor	Dilution
1	141Pr	Alpha-smooth muscle actin	14A	Standard BioTools; Maxpar® OnDemand™	1:300
2	147Sm	EpCAM	EPR20532-222	Standard BioTools; Maxpar® OnDemand™	1:150
3	150Nd	Ki-67	B56	Standard BioTools; Maxpar® OnDemand™	1:200
4	151Eu	CD45	D3F8Q	Standard BioTools; Maxpar® OnDemand™	1:200
5	152Sm	Fibronectin	EPR19241-46	Standard BioTools; Maxpar® OnDemand™	1:200
6	153Eu	CD44	IM7	Standard BioTools; Maxpar® OnDemand™	1:200
7	156Gd	F4/80	D2S9R	Standard BioTools; Maxpar® OnDemand™	1:200
8	158Gd	E-cadherin	24E10	Standard BioTools; Maxpar® OnDemand™	1:200
9	159Td	CD4	BLR167J	Standard BioTools; Maxpar® OnDemand™	1:200
10	160Gd	iNOS	SP126	Standard BioTools; Maxpar® OnDemand™	1:200
11	161Dy	MHC Class II	M5/114.15.2	Standard BioTools; Maxpar® OnDemand™	1:150
12	162Dy	CD8	EPR21769	Standard BioTools; Maxpar® OnDemand™	1:150
13	163Dy	CD11b	EPR1344	Standard BioTools; Maxpar® OnDemand™	1:300
14	165Ho	FoxP3	FJK-16s	Standard BioTools; Maxpar® OnDemand™	1:200
15	166Er	Ly-6G	1A8	Standard BioTools; Maxpar® OnDemand™	1:300
16	169Tm	Beta-catenin	5H10	Standard BioTools; Maxpar® OnDemand™	1:300
17	170Er	CD3	Polyclonal, C-terminal	Standard BioTools; Maxpar® OnDemand™	1:200
18	171Yb	CD31	EPR17259	Standard BioTools; Maxpar® OnDemand™	1:200
19	173Yb	Collagen Type I	Polyclonal	Standard BioTools; Maxpar® OnDemand™	1:300
20	174Yb	Pan-cytokeratin	AE-1/AE-3	Standard BioTools; Maxpar® OnDemand™	1:200
21	176Yb	B220	RA36B2	Standard BioTools; Maxpar® OnDemand™	1:400

Table S4. Cytometry by Time of Flight (CyTOF) antibody panel.

#	Label	Target (mouse)	Clone	Vendor	Catalog #	Dilution
1	89Y	CD45	30-F11	Standard BioTools	3089005B	1:125
2	106Cd	CD107a	M3/84	BioLegend; MCP9 Labelling	108502	1:100
3	110Cd	CD11b/Mac-1	M1/70	BioLegend; MCP9 Labelling	101202	1:100
4	111Cd	Ly6C	HK1.4	BioLegend; MCP9 Labelling	128039	1:100
5	113Cd	ARG1	W21047I	BioLegend; MCP9 Labelling	165802	1:100
6	141Pr	Ly-6G	1A8	Standard BioTools	3141008B	1:125
7	142Nd	CD11c	N418	Standard BioTools	3142003B	1:125
8	143Nd	CD69	H1.2F3	Standard BioTools	3143004B	1:125
9	144Nd	CD49b	HMα2	BioLegend; X8 Labelling	103513	1:100
10	145Nd	CD4	RM4-5	Standard BioTools	3145002B	1:125
11	146Nd	F4/80	BM8	Standard BioTools	3146008B	7:500
12	147Sm	TCR beta	H57-597	BioLegend; X8 Labelling	109202	1:100
13	148Nd	CD103	W19396D	BioLegend; X8 Labelling	110902	1:100
14	149Sm	CD19	6D5	Standard BioTools	3149002B	1:125
15	150Nd	IgD	11-26c.2a	Standard BioTools	3150011B	1:125
16	151Eu	CD25/IL-2R	3C7	Standard BioTools	3151007B	7:500
17	152Sm	CD3 epsilon	145-2C11	Standard BioTools	3152004B	1:125
18	153Eu	CD335/NKp46	29A1.4	Standard BioTools	3153006B	1:125
19	154Sm	TCR gamma delta	GL3	BioLegend; X8 Labelling	118101	1:100
20	156Gd	IL-33R	W17243F	BioLegend; X8 Labelling	165302	1:100
21	158Gd	FoxP3	FJK-16s	Standard BioTools	3158003A	1:50
22	159Tb	ROR gamma (t)	B2D	Standard BioTools	3159019B	1:125
23	160Gd	CD62L/L-selectin	MEL-14	Standard BioTools	3160008B	1:125
24	161Dy	IL-23R	12B2B64	BioLegend; X8 Labelling	150902	1:100
25	163Dy	CD170/Siglec-F	QA20A11	BioLegend; X8 Labelling	164102	1:100
26	164Dy	CX3CR1	SA011F11	Standard BioTools	3164023B	1:125
27	165Ho	IFNGR1/CD119	2E2	BioLegend; X8 Labelling	112802	1:100
28	166Er	CD16.2/FcÎ³RIV	9E9	BioLegend; X8 Labelling	149502	1:100
29	167Er	Gata3	TWAJ	Standard BioTools	3167007A	1:100
30	168Er	CD8a	53-6.7	Standard BioTools	3168003B	1:125
31	169Tm	CD206/MMR	C068C2	Standard BioTools	3169021B	1:125
32	170Er	CD161/NK1.1	PK136	Standard BioTools	3170002B	1:125
33	171Yb	CD80/B7-1	16-10A1	Standard BioTools	3171008B	1:125
34	172Yb	CD86	GL1	Standard BioTools	3172016B	1:125
35	174Yb	Tbet	4B10	BioLegend; X8 Labelling	644802	7:500
36	175Lu	SIRP1a/CD172a	QA20A06	BioLegend; X8 Labelling	108602	1:100
37	176Yb	CD45R/B220	RA3-6B2	Standard BioTools	3176002B	1:125
38	209Bi	MHC Class II	M5/114.15.2	Standard BioTools	3209006B	1:125

Table S5. Putative FlowSOM population annotations for CyTOF analysis of colon lamina propria (CLP).

**Colon lamina Propria (CLP)**		
**FlowSOM Population**	**Putative description**	**Phenotype**
Pop 5	γδ T cells	$CD45^{+}$ $TCR\gamma\delta^{+}$ $CD69^{hi}$ $CD3^{+}$
Pop 9	Type 2 conventional dendritic cells (cDC2s)	$CD45^{+}$ $CD11c^{+}$ $Ly6C^{-}$ $CD11b^{+}$ $MHCII^{hi}$ $SIRP\alpha^{+}$ $CD80^{+}$
Pop 15	Unclassified antigen presenting cells	$CD45^{+}$ $CD11b^{-}$ $CD4^{+}$ $MHCII^{+}$ $Ly6C^{+}$ $SIRP\alpha^{+}$
Pop 17	Newly tissue-infiltrating inflammatory monocytes	$CD45^{+}$ $Ly6C^{hi}$ $CD11b^{+}$ $MHCII^{-}$ $SIRP\alpha^{+}$ $CX3CR1^{+}$ $CD16.2^{+}$
Pop 18	Neutrophils	$CD45^{+}$ $CD11b^{hi}$ $Ly6G^{hi}$ $CD11c^{-}$ $SIRP\alpha^{+}$ CD16.2
Pop 19	Macrophage population with a reparative phenotype	$CD45^{+}$ $CD11c^{-}$ $Ly6C^{-}$ $CD11b^{+}$ $MHCII^{hi}$ $SIRP1\alpha^{+}$ $CD80^{+}$ $CD86^{+}$ $CX3CR1^{+}$ $Arg1^{hi}$ $CD206^{lo}$
